# Illness perception mediates the association between emotional intelligence and symptom distress among patients undergoing peritoneal dialysis: a Common-Sense Model approach

**DOI:** 10.3389/fpubh.2026.1885588

**Published:** 2026-08-04

**Authors:** Rong Zeng, Xiaoxia Lin, Xueqin Gan, Ting Luo, Min Li, Yuhang Chen, Qian Yang

**Affiliations:** 1 School of Nursing, Chengdu Medical College, Chengdu, Sichuan, China; 2 Department of Nephrology, The First Affiliated Hospital of Chengdu Medical College, Chengdu, Sichuan, China; 3 Department of Nursing, The Third People's Hospital of Sichuan Province, Chengdu, Sichuan, China

**Keywords:** emotional intelligence, illness perception, peritoneal dialysis, statistical mediation, symptom distress

## Abstract

**Background:**

This study aimed to examine the association between emotional intelligence and symptom distress among patients undergoing peritoneal dialysis (PD) based on the Common-Sense Model, and to explore whether illness perception statistically accounts for this association.

**Methods:**

A cross-sectional survey was conducted among 198 patients undergoing peritoneal dialysis in three tertiary hospitals in Chengdu, China. Emotional intelligence, illness perception, and symptom distress were assessed using validated questionnaires. Correlation and mediation analyses were performed to examine the associations among these variables.

**Results:**

The mean scores for the main variables were 79.22 ± 13.82 for emotional intelligence, 43.53 ± 7.88 for illness perception, and 24.04 ± 18.22 for symptom distress. Correlation analysis showed that emotional intelligence was significantly negatively related to both illness perception (*r* = −0.229, *p* < 0.001) and symptom distress (*r* = −0.234, *p* < 0.001). Illness perception was significantly positively related to symptom distress (*r* = 0.461, *p* < 0.001). Symptom distress differed significantly according to sex, employment status, monthly income, dialysis vintage, and the number of comorbidities. The total association between emotional intelligence and symptom distress was significant (*β* = −0.145, 95% CI [−0.285, −0.005]). After illness perception was included, the direct association was not significant (*β* = −0.079, 95% CI [−0.211, 0.053]), whereas the indirect association through illness perception was significant (*β* = −0.066, 95% bootstrap CI [−0.134, −0.007]). These findings suggest an indirect association within the cross-sectional statistical model rather than evidence of a causal pathway.

**Conclusion:**

Illness perception may be an important psychological correlate linking emotional intelligence with symptom distress in patients undergoing peritoneal dialysis. These findings highlight the potential value of integrating illness perception assessment and emotional regulation support into routine symptom management, although longitudinal and intervention studies are needed to clarify temporal relationships and evaluate clinical effectiveness.

## 1 Introduction

Peritoneal dialysis (PD) is one of the major home-based renal replacement therapies for patients with end-stage renal disease (1). Compared with in-center hemodialysis, PD offers greater flexibility and supports long-term survival; however, it also requires patients to undertake continuous self-management in their daily lives (2). During long-term treatment, patients may experience various dialysis-related symptoms and complications, such as fatigue, pain, sleep disturbance, gastrointestinal discomfort, fluid-related problems, and peritonitis-related concerns (3). These symptoms often occur repeatedly and may become an important source of physical and psychological burden among patients undergoing PD (4).

Symptom distress refers not only to the occurrence of physical symptoms but also to the degree to which these symptoms interfere with patients’ daily life, emotional state, and disease management (5). For patients undergoing PD, symptom distress may reduce quality of life, weaken treatment adherence, and increase negative emotional experiences such as anxiety and depression (6, 7). Because PD is mainly performed at home, patients are required to monitor symptoms, identify possible warning signs, and make daily decisions about fluid control, diet, medication, and dialysis procedures (2). Therefore, symptom distress in this population should not be understood only as a physiological problem; it is also closely related to patients’ psychological resources and their interpretation of illness-related experiences (8, 9).

Emotional intelligence is defined as the ability to perceive, understand, manage, and use emotions (10). In the context of chronic illness management, emotional intelligence may serve as an important psychological resource. Patients with higher emotional intelligence may be more capable of identifying negative emotions, regulating emotional responses to disease-related stress, and using adaptive coping strategies when facing long-term treatment demands (11). Previous studies have suggested that emotional intelligence is associated with better psychological adjustment and health-related outcomes in patients with chronic diseases (11, 12). Emotional intelligence may be particularly relevant in the context of PD because PD is largely performed at home and requires patients to assume substantial responsibility for daily treatment procedures, symptom monitoring, fluid control, dietary management, and early identification of complications (1, 2). Compared with institution-based dialysis care, patients undergoing PD frequently need to interpret bodily changes and make health-related decisions outside direct professional supervision. In this setting, the ability to recognize, understand, and regulate emotional responses may be closely related to how patients appraise illness-related threats and cope with symptom-related stress (10, 11, 13).

However, the association between emotional intelligence and symptom distress may not be direct. According to Leventhal’s Common-Sense Model, when individuals face a health threat, they form cognitive and emotional representations of the illness, known as illness perception (14). These representations include patients’ beliefs about the consequences, timeline, controllability, identity, and emotional impact of the disease, and they may further shape coping responses and health outcomes (15). In patients undergoing PD, illness perception may be related to how patients interpret dialysis-related symptoms and how much attention they pay to bodily discomfort (16). A more negative illness perception may amplify symptom experiences and increase symptom-related distress, whereas a more adaptive illness perception may help patients understand symptoms more accurately and cope with them more effectively (17, 18). In PD populations, illness perception has also been associated with self-management and coping style, suggesting its potential relevance to long-term disease management (19).

Within this framework, emotional intelligence may be associated with illness perception because patients with higher emotional intelligence may be better able to identify negative emotions, regulate dialysis-related anxiety, and reinterpret symptom-related information in a less catastrophic manner. Conversely, patients with lower emotional intelligence may be more likely to perceive bodily discomfort as threatening, uncontrollable, or unpredictable, thereby developing more negative illness representations. Therefore, illness perception may represent a cognitive-emotional component statistically linking emotional intelligence with symptom distress among patients undergoing PD.

Despite this theoretical rationale, several gaps remain. First, most previous studies in dialysis populations have focused on symptom prevalence, psychological distress, quality of life, or self-management, whereas the psychological association between emotional resources and symptom distress has received less attention (3, 4, 8). Second, although illness perception has been associated with coping and self-management in patients undergoing PD, limited evidence has examined whether illness perception statistically accounts for the association between emotional intelligence and symptom distress (13, 19). Third, few studies have integrated emotional intelligence, illness perception, and symptom distress within a Common-Sense Model framework in PD populations. Addressing this gap may provide a more integrated understanding of psychological correlates of symptom distress in patients undergoing PD.

**Figure 1:**
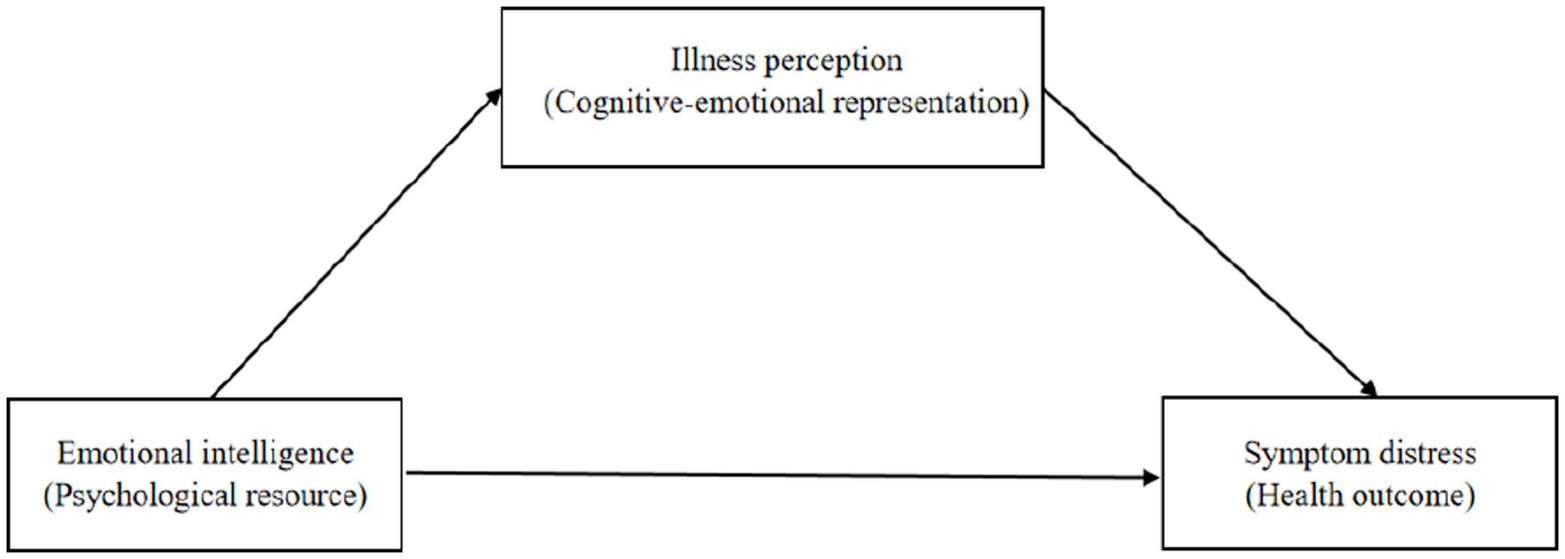
Theoretical framework based on the Common-Sense Model. The model illustrates the hypothesized indirect association among emotional intelligence, illness perception, and symptom distress within the Common-Sense Model framework.

Based on the Common-Sense Model, this study proposed a framework in which emotional intelligence was regarded as a psychological resource, illness perception as a cognitive-emotional representation, and symptom distress as a patient-reported health outcome (Figure 1) (14, 15). We hypothesized that emotional intelligence would be associated with symptom distress both directly and indirectly through illness perception. The originality of this study lies in examining these three constructs within a single CSM-based framework among patients undergoing PD, a population in which continuous home-based self-management makes the psychological interpretation of symptoms particularly important.

### 1.1 Purpose of the study

This study aimed to examine the associations among emotional intelligence, illness perception, and symptom distress in patients undergoing PD, and to explore whether illness perception statistically accounts for the association between emotional intelligence and symptom distress based on the Common-Sense Model.

## 2 Methods

### 2.1 Research design

This study employed a cross-sectional survey design based on Leventhal’s Common-Sense Model (CSM) to examine the associations among emotional intelligence, illness perception, and symptom distress among patients undergoing peritoneal dialysis (PD) (14, 15). Given the cross-sectional design, the analyses were intended to identify statistical associations and indirect association patterns rather than to establish causal relationships.

#### 2.1.1 Psychological resource component

Emotional intelligence was selected as the primary psychological resource factor, representing the individual’s ability to perceive, understand, manage, and utilize emotions (10). In the context of long-term PD treatment, emotional intelligence serves as a critical emotion-regulating resource that may help patients cope with psychological stress and may be associated with how they interpret disease threats.

#### 2.1.2 Cognitive-emotional representation component

Illness perception was operationalized as the cognitive-emotional representation factor within the CSM framework (14, 15). It reflects the patients’ subjective cognitive appraisal and emotional response to their disease and treatment. According to the CSM, illness perception may serve as a cognitive-emotional representation that links psychological resources with patient-reported health outcomes by altering the degree of attention to and amplification of symptoms.

#### 2.1.3 Health outcome component

Symptom distress was assessed as the primary health outcome. For PD patients, this encompasses the physical discomfort (such as fatigue and pain) and the accompanying negative emotional burden resulting from long-term dialysis (3, 4). Based on this theoretical framework, we tested the following hypotheses:

*H1*: Emotional intelligence is negatively associated with symptom distress among PD patients.

*H2*: Illness perception statistically accounts for the association between emotional intelligence and symptom distress.

This aligns with recent applications of the Common-Sense Model to chronic illness management and allows for a systematic examination of the ways in which emotional intelligence may be associated with symptom distress through illness perception within a cross-sectional statistical model (20, 21).

### 2.2 Data collection and study participants

The study participants were recruited using a convenience sampling method from the peritoneal dialysis centers of three tertiary grade A general hospitals in Chengdu, Sichuan Province, from January 2026 to April 2026. These centers provide regular follow-up and management for patients undergoing PD in routine clinical practice. However, because convenience sampling was used and all participating hospitals were located in Chengdu, the sample should be interpreted as a clinical convenience sample rather than a fully representative sample of all patients undergoing PD in China.

The sample size was determined using the formula for cross-sectional surveys: *n* = (*uα*/2 × *σ*/*δ*)^2^ (22). Based on a preliminary survey of 30 patients, the standard deviation (*σ*) of the total symptom distress score was 14.56. With *α* = 0.05 and an allowable error *δ* = 2.1, the minimum required sample size was calculated as 185. Accounting for a 10% dropout rate, the target sample size was set at 206. During data collection, all eligible patients approached agreed to participate, and no eligible patient refused participation. A total of 210 eligible patients agreed to participate and completed the questionnaires. Twelve questionnaires were excluded because of incomplete or invalid responses. Finally, 198 valid questionnaires were included in the analysis, with an effective response rate of 94.29%. No data imputation was performed because only complete and valid questionnaires were included in the final analysis. This study protocol was approved by the Ethics Committee of Chengdu Medical College (Approval no. CMCIR2025NO.053).

#### 2.2.1 Inclusion criteria

- Age ≥ 18 years.
- Diagnosed with end-stage renal disease (ESRD) and undergoing regular PD treatment for ≥3 months.
- Followed up at the PD center at least once every 4–8 weeks.
- Clear consciousness with normal understanding and communication abilities to independently complete the questionnaires.
- Provided informed consent and participated voluntarily.

#### 2.2.2 Exclusion criteria

- Patients who had changed their renal replacement therapy (e.g., switched to hemodialysis or received a kidney transplant).
- Concurrent severe conditions such as acute heart failure, severe respiratory infection, or malignant tumors that could confound symptom assessment.
- History of psychiatric disorders or severe cognitive impairment.

### 2.3 Instruments

#### 2.3.1 General characteristics

A researcher-designed general information questionnaire was utilized to collect participants’ demographic and clinical data. This included age, sex, education level, occupation, marital status, living arrangements, per capita monthly household income, medical payment methods, primary disease diagnosis, dialysis vintage, dialysis modality (continuous ambulatory peritoneal dialysis vs. automated peritoneal dialysis), residual urine volume, history of peritonitis, and the number of comorbidities.

#### 2.3.2 Peritoneal dialysis symptom distress scale (PDSDS)

The PDSDS, originally the Dialysis Symptom Index (DSI) developed by Weisbord et al. (23), which was later introduced and translated into Chinese by Zhou (24) and further developed and validated for patients undergoing PD (25), was used to assess the symptom burden of PD patients over the preceding week. The scale consists of 29 items covering common physical and emotional symptoms. For each symptom, patients first indicate its presence (Yes/No); if present, the level of distress is rated on a 5-point Likert scale ranging from 0 (“no impact”) to 4 (“extremely severe”). The total score ranges from 0 to 116, with higher scores reflecting a greater level of symptom distress. In the current study, the Cronbach’s *α* for this scale was 0.901.

#### 2.3.3 Wong Law emotional intelligence scale (WLEIS)

The WLEIS, developed by Wong and Law (26) and translated into Chinese by Wang et al. (27) was employed to measure patients’ emotional intelligence. The scale includes 16 items divided into four dimensions: self-emotion appraisal, others’ emotion appraisal, use of emotion, and regulation of emotion. Each item is rated on a 7-point Likert scale, from 1 (“strongly disagree”) to 7 (“strongly agree”). The total score ranges from 16 to 112, with higher scores indicating higher emotional intelligence. In this study, the Cronbach’s *α* for the WLEIS was 0.921.

#### 2.3.4 Brief illness perception questionnaire (BIPQ)

The BIPQ, developed by Broadbent et al. (28) and translated by Sun et al. (29) was used to assess patients’ cognitive and emotional representations of their illness. It comprises eight quantitative items assessing dimensions of cognitive representation (5 items), emotional representation (2 items), and illness comprehensibility (1 item). Each item is scored on a scale of 0–10, with the total score (0–80) calculated by summing the items. Higher scores signify a more negative or threatening perception of the disease. In the present study, the Cronbach’s *α* for this scale was 0.803.

The total BIPQ score was used to represent the overall level of negative or threatening illness perception. Although the BIPQ covers multiple dimensions, the present study focused on illness perception as an overall cognitive-emotional representation within the Common-Sense Model, rather than on the separate associations of individual dimensions. Using the total score also helped maintain model parsimony given the sample size and the mediation model structure.

### 2.4 Data analysis methods

Statistical analysis was performed using SPSS version 27.0 and the PROCESS macro (Model 4, 31). The specific analytical steps were as follows: First, frequency and percentage were used to describe categorical variables, while mean ± standard deviation was used for continuous variables. Second, independent-samples *t*-tests and one-way analysis of variance (ANOVA) were conducted to compare differences in symptom distress scores across demographic and clinical characteristics. Third, Pearson’s correlation analysis was performed to examine the associations among emotional intelligence, illness perception, and symptom distress. Fourth, potential common method bias was evaluated using both procedural controls and Harman’s single-factor test as a preliminary diagnostic approach (30). To reduce potential common method bias, anonymous questionnaire completion, voluntary participation, standardized instructions, and validated instruments were used during data collection. Harman’s single-factor test was interpreted cautiously because it is only a preliminary diagnostic method and cannot completely rule out common method bias. Fifth, the PROCESS macro (Model 4) proposed by Hayes (31) was used to examine whether illness perception statistically accounted for the association between emotional intelligence and symptom distress. A bootstrapping method with 5,000 resamples was applied, and an indirect association was considered statistically significant if the 95% confidence interval (CI) did not include zero. Covariates were selected by considering clinical relevance and the results of univariate analyses. Sex, employment status, per capita monthly household income, dialysis vintage, and number of comorbidities were included because they were clinically relevant to symptom distress and showed significant differences in the present sample. History of peritonitis was also examined as a clinically relevant PD-related variable. Because it was not significantly associated with symptom distress in the univariate analysis, it was not included in the primary adjusted model but was further examined in a sensitivity analysis. However, residual confounding from other theoretically relevant variables could not be fully excluded.

Before regression and mediation analyses, the assumptions of linear regression were examined. Normality of residuals was assessed using Q–Q plots and standardized residuals. Homoscedasticity was evaluated using scatterplots of standardized residuals against standardized predicted values. Multicollinearity was assessed using tolerance values and variance inflation factors (VIFs). All tests were two-tailed, with statistical significance set at *p* < 0.05.

## 3 Results

### 3.1 General characteristics of the participants

A total of 198 patients undergoing peritoneal dialysis (PD) were included in the final analysis. The demographic and clinical characteristics of the participants are summarized in Table 1. Of the 198 participants, 104 (52.53%) were male and 94 (47.47%) were female, with a mean age of 47.9 ± 13.9 years. The majority were married (75.25%), lived with their families (89.90%), and were currently unemployed (64.65%).

**Table 1:** Demographic and clinical characteristics of the participants and differences in symptom distress scores (*n* = 198).

Variables	Categories	*n* (%)	Symptom distress (mean ± SD)	*t*/*F*	*p*-value	Effect size
Sex	Male	104 (52.53)	21.20 ± 18.18	−2.332	0.021^*^	Cohen’s *d* = 0.33
	Female	94 (47.47)	27.18 ± 17.83			
Age (years)	18–29	19 (9.60)	20.16 ± 18.58	0.429	0.732	*η*^2^ = 0.007
	30–44	62 (31.31)	23.40 ± 18.21			
	45–59	72 (36.36)	24.78 ± 18.66			
	≥60	45 (22.73)	25.38 ± 17.70			
Education level	Primary school or below	34 (17.17)	28.91 ± 15.34	1.515	0.212	*η*^2^ = 0.023
	Junior high school	55 (27.78)	25.51 ± 20.48			
	Senior high/technical	47 (23.74)	22.19 ± 17.37			
	Junior college or above	62 (31.31)	21.47 ± 17.91			
Marital status	Unmarried	26 (13.13)	21.23 ± 17.10	0.835	0.435	*η*^2^ = 0.008
	Married	149 (75.25)	25.00 ± 17.91			
	Divorced/widowed	23 (11.62)	21.00 ± 21.32			
Employment status	Employed	70 (35.35)	20.49 ± 16.29	−2.047	0.042^*^	Cohen’s *d* = 0.30
	Unemployed	128 (64.65)	25.98 ± 18.97			
Household income	<3,000 RMB	101 (51.01)	26.40 ± 18.11	4.846	0.009^**^	*η*^2^ = 0.047
	3,000–5,000 RMB	59 (29.80)	25.19 ± 19.48			
	>5,000 RMB	38 (19.19)	16.00 ± 14.19			
Primary disease	Chronic glomerulonephritis	50 (25.25)	21.34 ± 17.86	2.39	0.052	*η*^2^ = 0.047
	Diabetic nephropathy	30 (15.15)	19.97 ± 15.44			
	Hypertensive glomerulosclerosis	50 (25.25)	29.28 ± 20.03			
	Polycystic kidney disease	6 (3.03)	35.33 ± 18.29			
	Other	62 (31.31)	22.87 ± 17.37			
Dialysis vintage	<12 months	67 (33.84)	21.79 ± 18.20	3.972	0.020^*^	*η*^2^ = 0.039
	13–36 months	55 (27.78)	20.55 ± 16.77			
	>36 months	76 (38.38)	28.55 ± 18.53			
Residual urine	<100 mL	62 (31.31)	27.90 ± 17.14	2.634	0.074	*η*^2^ = 0.026
	100–500 mL	57 (28.79)	24.23 ± 19.63			
	>500 ml	79 (39.90)	20.87 ± 17.60			
History of peritonitis	No	149 (75.25)	22.90 ± 18.12	−1.543	0.125	Cohen’s *d* = 0.25
	Yes	49 (24.75)	27.51 ± 18.26			
Comorbidities	1	77 (38.89)	21.57 ± 18.45	6.648	0.002^**^	*η*^2^ = 0.064
	2	72 (36.36)	21.24 ± 14.88			
	≥3	49 (24.75)	32.04 ± 20.21			

SD, standard deviation. Cohen’s *d* was reported for two-group comparisons, and *η*^2^ was reported for ANOVA comparisons. ^*^p < 0.05, ^**^p < 0.01.

Regarding clinical characteristics, the primary diagnoses were chronic glomerulonephritis (25.25%) and hypertensive glomerulosclerosis (25.25%). Most patients received continuous ambulatory peritoneal dialysis (CAPD) (92.93%) and had no history of peritonitis (75.25%). Univariate analysis showed that symptom distress scores differed significantly by sex (*t* = −2.332, *p* = 0.021, Cohen’s *d* = 0.33), employment status (*t* = −2.047, *p* = 0.042, Cohen’s *d* = 0.30), per capita monthly household income (*F* = 4.846, *p* = 0.009, *η^2^* = 0.047), dialysis vintage (*F* = 3.972, *p* = 0.020, *η^2^* = 0.039), and the number of comorbidities (*F* = 6.648, *p* = 0.002, *η^2^* = 0.064).

### 3.2 Common method bias test

To ensure the reliability of the self-reported data, Harman’s single-factor test was conducted prior to the main analysis to check for common method bias. The results showed a Kaiser–Meyer–Olkin (KMO) value of 0.789 and a significant Bartlett’s test of sphericity (*p* < 0.001). The unrotated principal component factor analysis extracted 15 factors with eigenvalues greater than 1. The variance explained by the first principal factor was 19.35%, which is well below the critical threshold of 40%. These results suggested that a single factor did not account for most of the variance. However, because Harman’s single-factor test is only a preliminary diagnostic method, common method bias could not be completely ruled out.

### 3.3 Scores and correlations among emotional intelligence, illness perception, and symptom distress

The descriptive statistics for the main variables and their correlations are presented in Table 2. The mean scores were 79.22 ± 13.82 for emotional intelligence, 43.53 ± 7.88 for illness perception, and 24.04 ± 18.22 for symptom distress.

**Table 2:** Descriptive statistics and correlation of variables.

Variables	M ± standard deviation	1r (*p*)	2r (*p*)	3r (*p*)
1. Emotional intelligence	79.22 ± 13.82	1		
2. Illness perception	43.53 ± 7.88	−0.229	1	
		(<0.001)		
3. Symptom distress	24.04 ± 18.22	−0.234	0.461	1
		(<0.001)	(<0.001)	

Pearson’s correlation analysis revealed that emotional intelligence was significantly and negatively correlated with illness perception (*r* = −0.229, *p* < 0.001) and symptom distress (*r* = −0.234, *p* < 0.001). Conversely, illness perception was significantly and positively correlated with symptom distress (*r* = 0.461, *p* < 0.001). These correlation results are summarized in Table 2.

### 3.4 Associations among emotional intelligence, illness perception, and symptom distress

To examine the associations among emotional intelligence, illness perception, and symptom distress, and to explore whether illness perception statistically accounted for the association between emotional intelligence and symptom distress, a bootstrap mediation analysis was conducted as proposed by Hayes (31). The PROCESS macro (Model 4) was applied with 5,000 bootstrap samples and 95% confidence intervals (CIs). Sex, employment status, per capita monthly household income, dialysis vintage, and the number of comorbidities were entered as covariates based on clinical relevance and the results of prior univariate analyses. Diagnostic checks indicated no serious violations of the major regression assumptions. The VIF values ranged from 1.060 to 1.160, and the tolerance values ranged from 0.862 to 0.944, indicating no serious multicollinearity among the predictors.

Table 3 presents the results of the regression analysis for each path in the mediation model. First, emotional intelligence showed a significant negative association with illness perception (*β* = −0.166, SE = 0.072, 95% CI [−0.309, −0.023], *p* < 0.05; *R*^2^ = 0.404, adjusted *R*^2^ = 0.381). Second, the total association between emotional intelligence and symptom distress was significant (*β* = −0.145, SE = 0.071, 95% CI [−0.285, −0.005], *p* < 0.05; *R*^2^ = 0.125, adjusted *R*^2^ = 0.084). Third, when emotional intelligence and illness perception were entered simultaneously, the direct association between emotional intelligence and symptom distress was not statistically significant (*β* = −0.079, SE = 0.067, 95% CI [−0.211, 0.053], *p* = 0.235), whereas illness perception demonstrated a significant positive association with symptom distress (*β* = 0.398, SE = 0.057, 95% CI [0.286, 0.510], *p* < 0.001; *R*^2^ = 0.227, adjusted *R*^2^ = 0.187). The total, direct, and indirect associations are presented in Table 4.

**Table 3:** Regression results for the statistical mediation model.

Path	*β*	SE	*t*	*p*-value	95% confidence interval	*R*^2^ (adj *R*^2^)
Emotional intelligence (X) → Illness perception (M)	−0.166	0.072	−2.292	<0.05	−0.309 to −0.023	0.404 (0.381)
Emotional intelligence (X) → symptom distress (Y) (total)	−0.145	0.071	−2.052	<0.05	−0.285 to −0.005	0.125 (0.084)
Emotional intelligence (X) → symptom distress (Y) (direct)	−0.079	0.067	−1.19	0.235	−0.211 to 0.053	0.227 (0.187)
Illness perception (M) → symptom distress (Y)	0.398	0.057	6.994	<0.001	0.286–0.510	–

*β*, standardized coefficient; SE, standard error; CI, confidence interval. Models were adjusted for sex, employment status, per capita monthly household income, dialysis vintage, and the number of comorbidities.

**Table 4:** Total, direct, and indirect associations among emotional intelligence, illness perception, and symptom distress.

Path	*β*	SE/boot SE	95% confidence interval (LLCI, ULCI)
Total association (X → Y)	−0.145	0.071	−0.285, −0.005
Direct association (X → Y)	−0.079	0.067	−0.211, 0.053
Indirect association (X → M → Y)	−0.066	0.032	−0.134, −0.007

*β*, standardized estimate; SE, standard error; CI, confidence interval. Confidence intervals for the total and direct associations were based on ordinary least-squares regression, whereas the confidence interval for the indirect association was estimated using 5,000 bootstrap samples. X = emotional intelligence; M = illness perception; Y = symptom distress.

**Figure 2:**
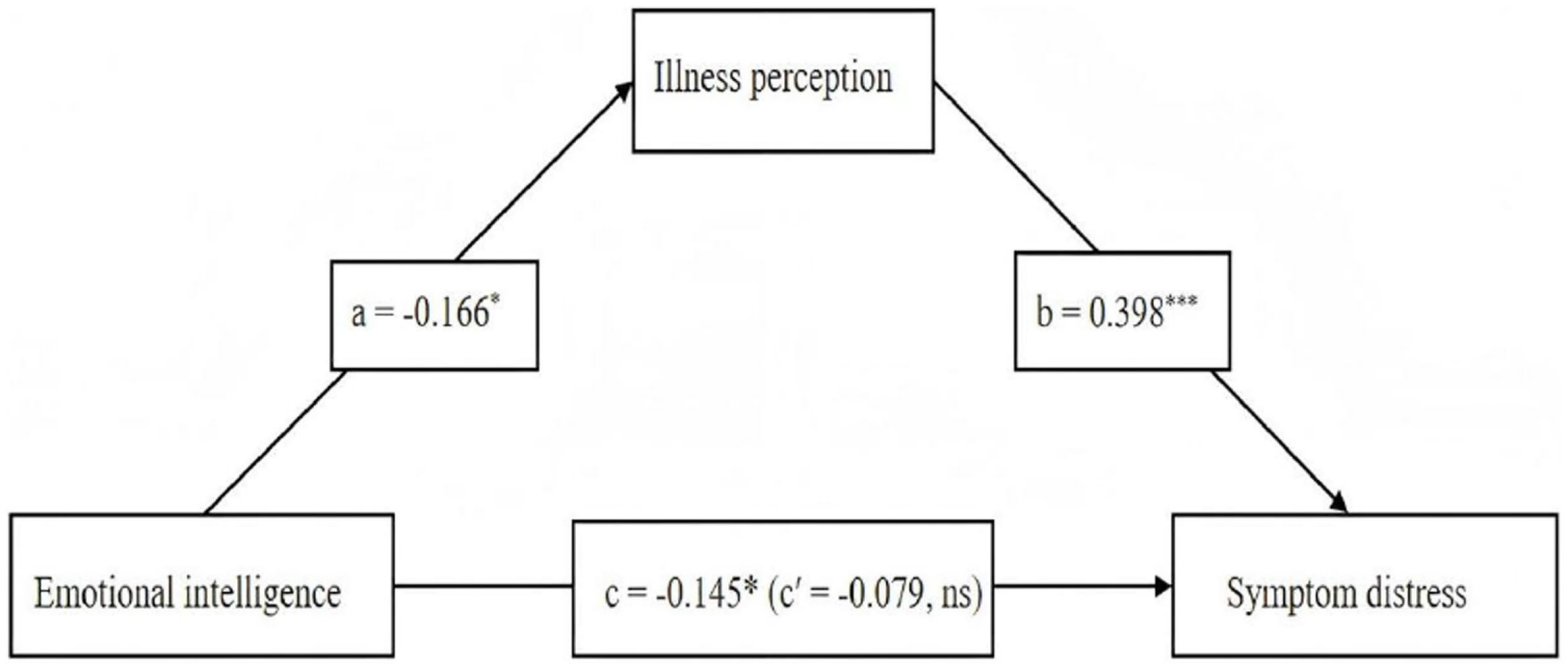
Cross-sectional statistical mediation model of the association between emotional intelligence and symptom distress through illness perception. Standardized path coefficients are presented. The value outside the parentheses represents the total association, and the value inside the parentheses represents the direct association after illness perception was included in the model. The indirect association was significant (*β* = −0.066, 95% bootstrap CI [−0.134, −0.007]). ^*^p < 0.05, ^***^p < 0.001; ns, not significant.

Based on the estimated path coefficients, the direct association between emotional intelligence and symptom distress was not statistically significant (*β* = −0.079, 95% CI [−0.211, 0.053]) (Table 4). In contrast, the standardized indirect association between emotional intelligence and symptom distress through illness perception was significant, as the 95% bootstrap CI did not include zero (*β* = −0.066, 95% bootstrap CI [−0.134, −0.007]). The indirect association accounted for 45.59% of the total association. Given the cross-sectional design, this finding should be interpreted as exploratory statistical evidence of an indirect association rather than confirmation of a causal mediation pathway. As a sensitivity analysis, history of peritonitis was additionally included as a covariate, and the main findings remained unchanged. The total association between emotional intelligence and symptom distress remained significant (*β* = −0.142, 95% CI [−0.283, −0.001]), the direct association remained non-significant (*β* = −0.079, 95% CI [−0.210, 0.052]), and the indirect association through illness perception remained significant (*β* = −0.063, 95% bootstrap CI [−0.134, −0.004]). The statistical mediation model is shown in Figure 2.

## 4 Discussion

This study examined the associations among emotional intelligence, illness perception, and symptom distress among patients undergoing peritoneal dialysis within the Common-Sense Model framework. The findings showed that emotional intelligence was negatively associated with illness perception and symptom distress, whereas illness perception was positively associated with symptom distress. In the cross-sectional statistical mediation model, the indirect association between emotional intelligence and symptom distress through illness perception was significant, while the direct association was not statistically significant after illness perception was included. These findings should be interpreted as preliminary evidence of an indirect association rather than confirmation of a causal mediation pathway.

### 4.1 Interpretation of the associations among emotional intelligence, illness perception, and symptom distress

The observed negative association between emotional intelligence and illness perception indicates that patients with higher emotional intelligence tended to report less threatening perceptions of their illness. This finding is clinically meaningful in the context of PD because patients are required to manage dialysis procedures, dietary restrictions, fluid control, and potential complications in their daily lives. Patients who are better able to identify, understand, and regulate their emotions may be more capable of interpreting disease-related changes in a less catastrophic manner (32, 33).

The positive association between illness perception and symptom distress further suggests that patients who perceived their illness as more threatening experienced greater symptom-related burden. According to the Common-Sense Model, illness perception reflects both cognitive appraisal and emotional response to a health threat. Negative illness perceptions may increase attention to bodily discomfort, amplify the perceived severity of symptoms, and weaken patients’ confidence in disease management (9, 34).

### 4.2 Differences in symptom distress according to demographic and clinical characteristics

Female patients, unemployed patients, and those with lower household income reported higher levels of symptom distress. These differences may reflect the combined influence of physiological, psychological, and socioeconomic vulnerability. Female patients may be more sensitive to physical discomfort and emotional changes during long-term treatment, while unemployment and lower income may increase financial pressure and reduce access to supportive resources (32, 35). For PD patients, treatment-related costs, lifestyle restrictions, and concerns about long-term prognosis may further intensify symptom-related distress (36, 37).

Patients with longer dialysis vintage and more comorbidities also showed higher symptom distress. This may be because prolonged dialysis exposure is accompanied by cumulative treatment burden, repeated symptom experiences, and increased risk of complications (38). Meanwhile, comorbid conditions may interact with dialysis-related symptoms, making it more difficult for patients to distinguish whether discomfort originates from PD itself, underlying kidney disease, or other chronic conditions (39).

These findings suggest that symptom management in PD patients should not rely solely on routine physiological assessment. Greater attention should be paid to patients with longer dialysis duration, multiple comorbidities, unstable employment status, and lower economic resources, as these groups may require more continuous symptom monitoring and psychosocial support (40).

### 4.3 Emotional intelligence and illness perception

In this study, emotional intelligence showed a significant negative association with illness perception, indicating that patients with higher emotional intelligence tended to hold less negative views of their illness. This result supports the idea that emotional intelligence is not only an emotion-related trait but also a psychological resource that shapes how patients interpret chronic illness. For patients undergoing PD, repeated exposure to symptoms, treatment procedures, and possible complications may easily lead to uncertainty and perceived loss of control. Higher emotional intelligence may help patients regulate anxiety, reframe disease-related information, and maintain a more balanced understanding of their condition (41, 42).

### 4.4 Indirect association through illness perception

When emotional intelligence and illness perception were entered into the mediation model simultaneously, the direct association between emotional intelligence and symptom distress was no longer statistically significant, whereas illness perception remained significantly associated with symptom distress. This pattern is consistent with an indirect association between emotional intelligence and symptom distress through illness perception within the cross-sectional statistical model. However, it should not be interpreted as evidence of a causal mediation process. In other words, the association between emotional intelligence and symptom distress may reflect the interplay between emotional processing and illness-related cognitive appraisal, in which emotional regulation capacity and illness perception operate as intertwined and complementary psychological facets rather than as separate or exclusive processes.

This finding is consistent with the Common-Sense Model, which emphasizes that patients’ cognitive and emotional representations of illness influence coping responses and health outcomes. In the context of PD, symptom distress is not determined only by objective physical symptoms. Patients’ interpretation of these symptoms, their perceived control over the disease, and their emotional responses to long-term dialysis may also play important roles. Therefore, illness perception may function as a possible cognitive-emotional link between emotional regulation resources and symptom-related outcomes (9, 34).

From a theoretical perspective, these findings may extend the application of the Common-Sense Model in patients undergoing PD by linking emotional regulation resources with illness representations and symptom-related outcomes. In the traditional CSM framework, illness perception is often viewed as patients’ cognitive and emotional representation of a health threat. The present findings suggest that emotional intelligence may be associated with how patients form or maintain these illness representations, while illness perception may further be associated with symptom distress. Thus, this study provides a CSM-based perspective for understanding how emotional resources and illness representations may be jointly related to symptom distress in patients undergoing PD. However, given the cross-sectional design, this theoretical interpretation should be regarded as a plausible explanation rather than evidence of a causal sequence.

Alternative explanations should also be considered. Although the statistical model was based on the Common-Sense Model, the cross-sectional design does not allow determination of temporal order. Patients with higher symptom distress may become more attentive to bodily discomfort, develop more threatening illness perceptions, and experience greater emotional difficulty. In addition, unmeasured factors such as anxiety, depression, health literacy, family support, disease severity, treatment burden, and healthcare access may also contribute to the associations among emotional intelligence, illness perception, and symptom distress. Therefore, the observed indirect association should be interpreted as one possible statistical explanation rather than evidence of a confirmed psychological mechanism.

### 4.5 Clinical implications and intervention considerations

Emotional intelligence should be interpreted carefully in the clinical context. It may reflect both relatively stable individual differences and potentially modifiable emotional skills. Some aspects of emotional intelligence, such as emotional awareness and general emotion-related tendencies, may be relatively stable. However, specific skills, including recognizing negative emotions, regulating emotional responses, and reappraising illness-related stress, may be strengthened through psychological education, cognitive-behavioral strategies, mindfulness-based approaches, or nurse-led supportive interventions. Therefore, emotional intelligence should not be viewed only as a fixed trait. Nevertheless, whether improving emotional regulation capacity can modify illness perception or reduce symptom distress among patients undergoing PD requires further evaluation in longitudinal and intervention studies.

These findings may have implications for symptom management in patients undergoing PD, but they should be applied cautiously. Healthcare professionals may consider incorporating assessment of emotional regulation capacity and illness perception into routine follow-up, rather than focusing only on physical symptoms. Such assessment may help identify patients who perceive PD as highly threatening, uncontrollable, or emotionally overwhelming.

From an intervention perspective, emotional regulation support and illness-perception-focused cognitive reappraisal may be considered complementary rather than independent targets. Future interventions could consider integrating emotional regulation support, cognitive reappraisal strategies, illness education, symptom interpretation guidance, and self-management support to help patients process emotional responses and reinterpret dialysis-related symptoms in a more adaptive manner. For example, nurses could help patients identify negative emotional responses, reinterpret dialysis-related symptoms, and distinguish expected treatment-related discomfort from warning signs requiring medical attention. Tailored supportive strategies may be particularly relevant for patients reporting higher symptom distress, especially those with longer dialysis vintage, multiple comorbidities, lower income, unemployment, or other vulnerability factors. However, the present cross-sectional findings do not establish that modifying emotional intelligence or illness perception will directly reduce symptom distress. Future longitudinal and intervention studies are needed to evaluate the effectiveness of such programs.

### 4.6 Cultural context

Cultural context should also be considered when interpreting these findings. This study was conducted among Chinese patients undergoing PD, and illness perception and emotional expression may be influenced by sociocultural norms. In Chinese cultural settings, family involvement, concerns about being a burden to others, respect for medical authority, and relatively restrained emotional expression may affect how patients understand illness, communicate symptoms, and report emotional distress. These factors may also shape how patients appraise dialysis-related symptoms and seek support during long-term home-based treatment. Therefore, the associations among emotional intelligence, illness perception, and symptom distress observed in this study may not be directly generalizable to patients in different cultural or healthcare contexts. Future cross-cultural studies are needed to examine whether similar patterns exist in other populations.

### 4.7 Limitations and suggestions for future research

This study has several limitations. First, the cross-sectional design prevents causal inference regarding the relationships among emotional intelligence, illness perception, and symptom distress. Future longitudinal or intervention studies are needed to clarify the temporal sequence and directional relationships among these variables. In addition, although several clinically relevant covariates were adjusted for in the mediation model, residual confounding from unmeasured or unadjusted factors could not be fully excluded. Future studies with larger samples should consider a broader range of potential confounders, including additional sociodemographic, clinical, and psychosocial variables. Second, all data were collected using self-reported questionnaires, which may have introduced recall bias or social desirability bias. Future studies could combine self-reported data with clinical indicators, symptom diaries, or qualitative interviews to obtain a more comprehensive understanding of patients’ symptom experiences. Third, participants were recruited from three tertiary hospitals in Chengdu using convenience sampling, which may limit the generalizability of the findings. Future multicenter studies with larger and more diverse samples are needed to validate the present results. Finally, this study focused mainly on individual psychological factors. Future research could incorporate family support, healthcare access, treatment adherence, and clinical indicators to build a more comprehensive model of symptom distress among PD patients.

## 5 Conclusions and recommendations

This study examined the associations among emotional intelligence, illness perception, and symptom distress in patients undergoing peritoneal dialysis. The findings showed that higher emotional intelligence was associated with less negative illness perception and lower symptom distress, whereas more negative illness perception was associated with greater symptom distress. The indirect association between emotional intelligence and symptom distress through illness perception was statistically significant within the cross-sectional mediation model. However, given the cross-sectional design, these findings should be interpreted as preliminary evidence of an indirect association rather than confirmation of a causal mediation pathway.

These findings suggest that illness perception may be an important psychological correlate linking emotional intelligence with symptom distress in patients undergoing peritoneal dialysis. In clinical practice, healthcare professionals may consider incorporating assessment of illness perception and emotional regulation capacity into routine symptom management. Such assessment may help identify patients who report more threatening illness perceptions or greater emotional difficulty when coping with dialysis-related symptoms.

Future longitudinal and intervention studies are needed to clarify the temporal relationships among emotional intelligence, illness perception, and symptom distress. Intervention studies could further evaluate whether programs integrating emotional regulation training, cognitive reappraisal, illness education, and self-management support can improve illness perception and reduce symptom distress among patients undergoing peritoneal dialysis.

## Data Availability

The raw data supporting the conclusions of this article will be made available by the authors, without undue reservation.
